# Interrelationships between sex and dietary lysine on growth performance and carcass composition of finishing boars and gilts^1^

**DOI:** 10.1093/tas/txaa129

**Published:** 2020-07-18

**Authors:** Pau Aymerich, Carme Soldevila, Jordi Bonet, Mercè Farré, Josep Gasa, Jaume Coma, David Solà-Oriol

**Affiliations:** 1 Vall Companys Group, Lleida, Spain; 2 Animal Nutrition and Welfare Service, Department of Animal and Food Sciences, Universitat Autònoma de Barcelona, Bellaterra, Spain; 3 $Department of Mathematics, Area of Statistics and Operations Research, Universitat Autònoma de Barcelona, Bellaterra, Spain

**Keywords:** boars, finishing pig, gilts, lysine, requirements

## Abstract

The main goals of this study were to determine whether boars and gilts respond differently to the standardized ileal digestible lysine to net energy ratio (SID Lys:NE) and model the response to optimize growth performance. A total of 780 finishing pigs, 390 boars and 390 gilts [Pietrain NN × (Landrace × Large White)], with an initial individual body weight of 70.4 ± 9.2 for boars and 68.7 ± 8.0 kg for gilts, were used in a 41-d dose–response experiment. Pens (13 pigs per pen) were randomly allocated to a dietary treatment (2.64, 3.05, 3.46, 3.86, 4.27 g SID Lys/Mcal NE) by block and sex, with six replicates per treatment and sex. Two isoenergetic diets (2,460 kcal NE/kg), representing the extreme SID Lys:NE, were formulated and then mixed. Pigs were individually weighed at days 0, 22, and 41, when the experiment finished. The differential effect of SID Lys:NE on growth performance and carcass composition between sexes was analyzed with orthogonal polynomial contrasts to compare the linear and quadratic trends in each sex. In addition, broken-line linear (BLL) models to optimize average daily gain (ADG), including average daily feed intake (ADFI) as a covariate, were fitted when possible. As expected, boars had a greater ADG and feed efficiency (G:F; *P* < 0.001) than gilts, but there was no evidence of differences in ADFI (*P* = 0.470). Increasing SID Lys:NE had a greater linear impact on boars ADG (*P* = 0.087), G:F (*P* = 0.003), and carcass leanness (*P* = 0.032). In contrast, gilts showed a greater linear increase in SID Lys intake per kg gain (*P* < 0.001) and feed cost per kg gain (*P* = 0.005). The best fitting BLL models showed that boars maximized ADG at 3.63 g SID Lys/Mcal NE [95% confidence interval (CI): (3.32 to 3.94)], although another model with a similar fit, compared with the Bayesian information criterion, reported the optimum at 4.01 g SID Lys/Mcal NE [95% CI: (3.60, 4.42)]. The optimum to maximize ADG for gilts was estimated at 3.10 g SID Lys/Mcal NE [95% CI: (2.74, 3.47)]. Thus, the present study confirmed that boars and gilts have a different linear response to SID Lys:NE, explained by the greater protein deposition potential of boars. Likewise, BLL models indicated that boars require a higher SID Lys:NE to maximize ADG from 70 to 89 kg. These results suggest that split feeding of finishing boars and gilts could be beneficial in terms of both performance and cost return.

## INTRODUCTION

Historically, entire male pig production was common in some European countries such as Spain, the British Isles, and Portugal (Bee et al., 2015). However, with the increasing pressure to ban castration in the EU and as a result of the *Council Directive 2008/120/EC*, in 2017 boars already accounted for 34% of the EU male pig population (Kress et al., 2019), representing around 45 million pigs (Agri-Food Data Portal—Pigmeat Productions; agridata.ec.europa.eu). As this has been a recent change, most of the studies conducted to determine lysine requirements for growth performance used barrows instead of boars (Szabó et al., 2001; Ettle et al., 2003; Main et al., 2008; Elsbernd et al., 2017). During recent decades, some works have already studied the differential response of boars compared with gilts (Campbell et al., 1988; Van Lunen and Cole, 1996; King et al., 2000; O’Connell et al., 2006; Moore et al., 2013), whereas others focused on the differences as a result of castration (Williams et al., 1984; Otten et al., 2013; Moore et al., 2016). In addition, there have been some modeling studies that compared lysine requirements based on growth and feed intake data (Quiniou et al., 2010; NRC, 2012; van der Peet-Schwering and Bikker, 2018).

Boars are known to have a greater potential for growth than gilts from 40 to 70 kg body weight (BW) until market weight (Campbell et al., 1988; Van Lunen and Cole, 1996; Moore et al., 2013; Cámara et al., 2014), resulting in a leaner body and carcass composition (Andersson et al., 2005; Giles et al., 2009; Gispert et al., 2010; Aymerich et al., 2019). As no evidence of differences in feed intake has been reported in the literature (Van Lunen and Cole, 1996; O’Connell et al., 2006; Moore et al., 2013; Cámara et al., 2014), theoretically the greater protein deposition potential of boars would represent a greater lysine requirement to maximize average daily gain (ADG). Most studies have found differences in requirements between boars and gilts in the finishing phase (Campbell et al., 1988; O’Connell et al., 2006); however, some studies have not (Van Lunen and Cole, 1996; King et al., 2000).

The available literature lacks research that compares the lysine requirements of boars and gilts in commercial conditions using low feed intake sire-lines, such as Pietrain. If, as hypothesized, boars have greater SID Lys:NE requirements than gilts, it might be productively and economically worthwhile to split-feed pigs by sex (Coffey et al., 1995) or use precision feeding systems (Pomar et al., 2010). Thus, the present work studied the effect of SID Lys:NE on the growth performance (70 to 106 kg) and carcass composition of finishing boars and gilts. The objectives of this work were 1) to determine whether boars and gilts respond differently to the standardized ileal digestible lysine to net energy ratio (SID Lys:NE) and 2) to model the response to SID Lys:NE in order to determine the SID Lys:NE requirement to maximize performance.

## MATERIALS AND METHODS

All the procedures described in this work followed the EU Directive 2010/63/EU for animal experiments.

### Experimental Design and Animals

In this study, the differential response between finishing boars and gilts to increasing SID Lys:NE was analyzed in a 41-d dose–response experiment with five increasing levels. The trial was conducted in a commercial-experimental farm integrated into Vall Companys Group (Alcarràs, Spain). The study sample consisted of a total of 780 finishing pigs, 390 boars and 390 gilts [Pietrain NN × (Landrace × Large White)], with an initial individual BW of 70.4 ± 9.2 for boars and 68.7 ± 8.0 kg (mean ± SD) for gilts. When the pigs entered the growing-finishing facilities, they were separated into pens (13 pigs per pen) of similar weight based on visual observation. They were then weighed, and large pigs that were in pens classified as small were exchanged with small pigs in pens classified as large, and vice versa, until we had three BW blocks (19.1 ± 2.6, 21.9 ± 2.6, and 24.7 ± 2.6 kg, for small, medium, and large categories, respectively). At the start of the experiment, the pens were randomly allocated by block to each treatment, and the resulting distribution was checked to avoid confounding effects related to barn location. Pen was used as an experimental unit, with six replicates per treatment and sex. Each pen had a half slatted concrete floor (3 × 3 m), one-hole wet–dry Maxi Grow Feeder (Rotecna, Agramunt, Spain), and an additional nipple waterer at the opposite side. The farm was both naturally and semi-forced ventilated. Natural light was provided through the windows used for ventilation, and artificial light was only used when required by the farm care personnel. Although the trial was conducted during winter, the underfloor heating system was only used on the first days of the growing period, before starting the trial. Ad libitum access to feed and water was ensured during the entire trial. Pigs were individually weighed and monitored using electronic ear tags at the beginning, at day 22 and before marketing the heaviest pigs (day 41 of the trial). The pigs came from a healthy sow farm, and no relevant health issues were observed during the experiment. At day 41 of the experiment, pigs in the heaviest BW block were moved to the slaughterhouse (Patel S.A.U; L’Esquirol, Spain), and individual carcass composition was measured with AutoFom III (Frontmatec Food Technology, Kolding, Denmark). Measured parameters included hot carcass weight (HCW; head and feet on), carcass leanness (CL), backfat thickness (BFT), and ham fat thickness (HFT), whereas carcass yield (CY) was calculated afterwards. The same procedure was performed on medium and small BW block pigs at days 48 and 55, respectively.

### Feeding

Two isoenergetic diets (2,460 kcal NE/kg) were formulated based on maize, wheat, and soybean meal (Table 1). Diets were formulated to meet or exceed the requirements for each nutrient, except lysine. The essential amino acid (AA) was formulated based on the ideal protein ratios (NRC, 2012; FEDNA, 2013). The high ratio diet was 4.27 g SID Lys/Mcal NE (Feed A), and the low ratio diet was 2.64 g SID Lys/Mcal NE (Feed B). To reduce SID Lys:NE, the amount of soybean meal included was decreased and the amount of wheat increased. In addition, the amount of crystalline AA included was also reduced ensuring that the ideal protein ratios were met in both diets. The two extreme feeds were mixed in five different proportions (Table 2) in the farm using a robotic feeding system (DryExact Pro; Big Dutchman, Vechta, Germany). Feed intake was measured weekly by determining the amount of feed remaining in each trough.

**Table 1.** Ingredient composition and calculated nutritional composition of the two diets used for blending the five dietary treatments (as-fed basis)

Ingredient composition, %	Feed A	Feed B	Calculated composition^1^	Feed A	Feed B
Maize	45.00	45.00	Dry matter, %	87.56	87.42
Wheat	34.13	39.51	Crude fiber, %	2.76	2.74
Soybean meal 47%	15.20	11.20	Sugars, %	2.68	2.54
Animal fat	1.60	1.50	Starch, %	47.90	51.03
Calcium carbonate	1.20	1.20	Ether extract, %	3.75	3.66
Dicalcium phosphate	0.20	0.20	Crude protein, %	15.69	13.55
Sodium chloride	0.40	0.40	Total Lys, %	1.14	0.73
Lysine sulphate	0.91	0.33	SID Lys, %	1.05	0.65
l-Threonine	0.27	0.04	SID Met+ Cys/Lys ratio	0.61	0.62
dl-Methionine	0.22	—	SID Thr/Lys ratio	0.66	0.65
l-Valine	0.12	—	SID Trp/Lys ratio	0.21	0.21
l-Tryptophan	0.07	—	SID Val/Lys ratio	0.65	0.79
l-Isoleucine	0.06	—	SID Ile/Lys ratio	0.55	0.69
Phytase^2^	0.02	0.02	ME, kcal/kg	3,268	3,257
Acids mix^3^	0.30	0.30	NE, kcal/kg	2,460	2,460
Vit–min premix^4^	0.30	0.30	SID Lys:NE ratio, g/Mcal	4.27	2.64
			Ashes, %	3.74	3.57
			Total Ca, %	0.58	0.57
			STTD Ca, %	0.45	0.44
			Total P, %	0.36	0.35
			STTD P, %	0.30	0.30
			Cl, %	0.28	0.28
			K, %	0.58	0.52
			Na, %	0.16	0.16

^1^SID = standardized ileal digestible; ME = metabolizable energy; NE = net energy; STTD = standardized total tract digestible. ^2^6-Phytase (750 FTU/kg). ^3^Blend of formic and lactic acid. ^4^Provided per each kg of complete feed: 4,500 IU vitamin A, 2,000 IU vitamin D_3_, 15 mg vitamin E, 0.7 mg vitamin K, 1.0 mg vitamin B_1_, 4.0 vitamin B_2_, 1.2 vitamin B_6_, 0.02 vitamin B_12_, 15 mg niacin, 12 mg pantothenic acid, 107 mg coline, 90 mg Fe from iron sulphate, 100 mg Zn from zinc sulphate, 50 mg Mn from manganese oxide, 20 mg Cu from copper sulphate, 1.8 mg I from potassium iodide and 0.25 mg Se from sodium selenite.

**Table 2.** Blend ratios, calculated composition, and price for the five dietary treatments (as-fed basis)

Item^1^	T1	T2	T3	T4	T5
Feed A, %	0	25	50	75	100
Feed B, %	100	75	50	25	0
SID Lys, %	0.65	0.75	0.85	0.95	1.05
NE, kcal/kg	2,460	2,460	2,460	2,460	2,460
SID Lys:NE, g/Mcal	2.64	3.05	3.46	3.86	4.27
Formula cost, €/t^2^	223.4	231.0	238.5	246.1	253.6

^1^SID = standardized ileal digestible; NE = net energy. ^2^Calculated as a weighted average of the formula cost and inclusion of Feeds A and B.

### Diet Sampling and Analysis

After pelleting, feed samples were collected for each successive blending batch (5,000 kg), and crude protein (CP) was analyzed (ISO 16634-2:2016) to ensure that CP was within the range of the calculated composition. In addition, AA composition (Method 994.12, AOAC International, 2007) was analyzed in a blend of all the different batches (Table 3).

**Table 3.** Analyzed and calculated amino acid content of the experimental feeds (A and B) used for blending and obtain the five dietary treatments (%, as-fed basis)

Item	Feed A		Feed B	
	Calculated	Analyzed	Calculated	Analyzed
Crude protein	15.7	15.9	13.6	13.7
Lysine	1.14	1.14	0.73	0.77
Methionine	0.44	0.40	0.21	0.21
Methionine + cysteine	0.71	0.65	0.47	0.45
Threonine	0.78	0.76	0.50	0.53
Valine	0.78	0.78	0.60	0.61
Leucine	1.21	1.21	1.11	1.10
Isoleucine	0.63	0.64	0.51	0.52
Histidine	0.38	0.37	0.35	0.34
Arginine	0.87	0.90	0.76	0.80

### Calculations and Statistical Analyses

Pen BW, ADG, average daily feed intake (ADFI), feed efficiency (G:F), g SID Lys daily intake, g SID Lys intake per kg gain, and feed cost per kg gain were measured and calculated for *Period 1* (days 0 to 22), *Period 2* (days 22–41), and *Overall* (days 0 to 41). In addition, HCW, CY, CL, BFT, and HFT were calculated per pen using the individual data from pigs that could be traced at the slaughterhouse. The effect of SID Lys:NE on the studied productive parameters was initially analyzed in a linear model including SID Lys:NE, sex, initial BW block, and all the interactions between factors as fixed effects. Interactions that were not significant (*P* > 0.050) and/or biologically meaningless for the *Overall* period were not included in the final model because simplification was prioritized. As not all pigs could be completely traced at the slaughterhouse, the number of pigs per pen was included as a weighting factor in the carcass trait models. In addition, CY, CL, BFT, and HFT were linearly adjusted using HCW as a covariate. The differential effect of SID Lys:NE on growth performance between sexes was analyzed implementing orthogonal polynomial contrasts to compare the linear and quadratic trends in each sex. Afterwards, the model was conditioned to determine the linear or quadratic effect of SID Lys:NE on each sex. The models were performed using the *stats* package (R Core Team, 2019), ANOVA with the *car* package (Fox and Weisberg, 2019), and the effect of SID Lys:NE was contrasted using the *emmeans* package (Lenth, 2020).

Broken-line linear (BLL) regression models were fitted when possible to determine the break point at which ADG was maximized for each sex using the *stats* package (R Core Team, 2019). The models used were adapted from Robbins et al. (2006) by including ADFI as a covariate to improve the predictability of the model:

$$\begin{array}{l} Y_{ij}=L+U*\;\;\;(R-X_{i})+\pi *Z_{ij}+e_{ij} \end{array}$$

if $X_{i}\leq R$, and when $X_{i}>R$ then

$$\begin{array}{l} Y_{ij}=L+\pi *Z_{ij}+e_{ij} \end{array}$$

where $Y_{ij}$ is the response in ADG as a result of the difference between the break point (*R*) and SID Lys:NE (*X*_*i*_) times a factor (*U*) before reaching the plateau (*L*). In both equations, we assumed a linear effect (*π*) of ADFI (*Z*_*ij*_) on the dependent variable. The models reported in the results section are the best fitting models achieved by iteratively modifying the initial parameter values and selecting the model with a lower Bayesian information criteria (BIC). Finally, the 95% confidence interval (CI) for the BLL parameters (Venables and Ripley, 2002) was estimated with the *nlstools* package (Baty et al., 2015) to compare the requirements of boars and gilts. For all tests, results were considered significant when *P* ≤ 0.05, and a tendency when 0.05 < *P* ≤ 0.10.

## RESULTS

**Figure 1.**
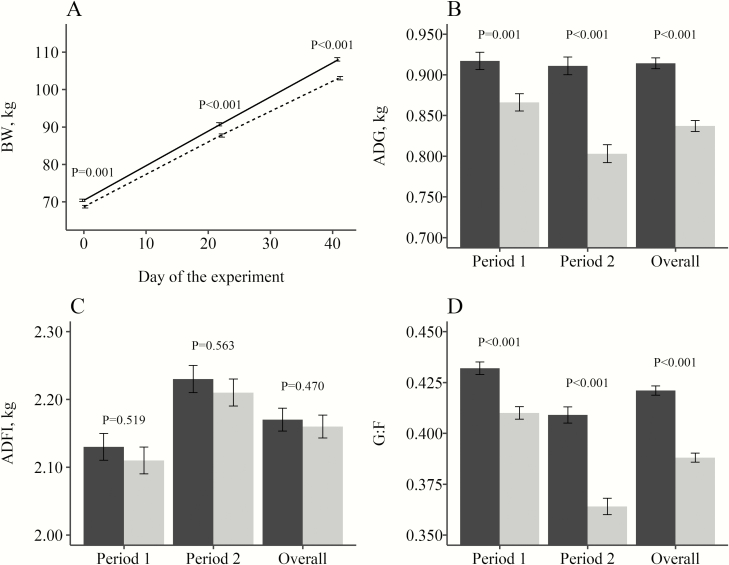
Effect of sex on body weight (A), average daily gain (B), average daily feed intake (C), and gain to feed (D) of finishing boars and gilts. For A, data represent the three weighing days. For B, C and D results are provided for Period 1 (70 to 89 kg), Period 2 (89 to 106 kg) ,and the overall experiment (70 to 106 kg). Solid line and black bars represent boars, whereas dashed line and gray bars represent gilts. Error bars represent the SEM.

The analyzed protein and AA content of the experimental feeds (Table 3) were consistent with the calculated composition of the diets. *Overall*, there was no evidence of significant interactions (*P* > 0.050) between block and treatment or sex. Only a tendency for an interaction between block and SID Lys:NE (*P* = 0.051) was observed for SID Lys per kg gain; however, as it represented a different effect only in medium category pigs, it was not considered biologically meaningful. Therefore, a model including only the interaction between SID Lys:NE and sex was preferred for the sake of simplification. An overview of the main effects of sex is presented in Fig. 1 for the main growth performance variables. Initially boars weighed 1.7 kg more than gilts (70.4 vs. 68.7 kg, *P* = 0.001), but this difference was even greater at day 22 (90.7 vs. 87.7 kg, *P* < 0.001) and at the end of the trial (108.0 vs. 102.9 kg, *P* < 0.001). The increase in the difference in BW throughout the experiment was the result of a greater ADG of boars during *Period 1* (*P* = 0.001) and *Period 2* (*P* < 0.001). No evidence of differences in ADFI between boars and gilts was observed (*P* > 0.100), and therefore the greater growth was considered the result of an increased G:F of boars (*P* < 0.001). The *Overall* results reported a greater ADG (0.914 vs. 0.837 kg, *P* < 0.001) and G:F (0.421 vs. 0.388, *P* < 0.001) of boars, but no evidence of a difference in ADFI (2.17 vs. 2.16 kg, *P* = 0.470). As expected, the improved performance of boars was also reflected in a leaner carcass composition. Hot carcasses of boars were heavier (89.7 vs. 86.6 kg, *P* < 0.001), and CY was greater for gilts (77.2 vs. 78.7%, *P* < 0.001). Boars had greater CL (64.4 vs. 63.5%, *P* < 0.001) and lower BFT (14.1 vs. 14.7 mm, *P* = 0.009) and HFT (8.82 vs. 10.29 mm, *P* < 0.001).

### Differential Response to SID Lys:NE

In this section, the comparison of the linear and quadratic trends depending on SID Lys:NE between sexes is provided and used to determine whether there was a differential response between sexes. In addition, the effect of SID Lys:NE on each sex is also provided. Growth performance results of *Period 1* (70 to 89 kg) are summarized in Table 4. Strong evidence of a different linear response to SID Lys:NE was found for some performance variables. Nevertheless, there was no evidence of a different quadratic response to SID Lys:NE between boars and gilts for any of the variables analyzed. Regarding ADG, linear trends between sexes when SID Lys:NE was increased were not significantly different (*P* = 0.115). Nevertheless, boars showed a linear increase (*P* < 0.001), but gilts just a tendency (*P* = 0.100). The ADFI of both boars and gilts was not linearly or quadratically affected by SID Lys:NE. Although G:F increased linearly when SID Lys:NE was increased in both boars (*P* < 0.001) and gilts (*P* = 0.005), boars showed a greater linear increase than gilts (*P* = 0.003). Furthermore, there was a greater linear increase in SID Lys/kg gain (*P* = 0.001) and in feed cost per kg gain (*P* = 0.004) when SID Lys:NE was increased for gilts compared with boars.

**Table 4.** Interactive effects between standardized ileal digestible lysine to net energy ratio (SID Lys:NE) and sex on growth performance and economics of finishing boars and gilts in Period 1 (70 to 89 kg)^1^

Item		SID Lys:NE, g/Mcal^2^					SEM	*P*-value			
		2.64	3.05	3.46	3.86	4.27		L × S^3^	Q × S^4^	Linear	Quadratic
ADG, kg	Boars	0.830	0.918	0.934	0.934	0.968	0.0235	0.115	0.626	<0.001	0.168
	Gilts	0.823	0.882	0.857	0.882	0.885				0.100	0.483
ADFI, kg	Boars	2.11	2.21	2.15	2.08	2.10	0.048	0.579	0.832	0.288	0.356
	Gilts	2.10	2.14	2.11	2.11	2.09				0.776	0.532
G:F	Boars	0.394	0.416	0.436	0.452	0.461	0.0072	0.003	0.612	<0.001	0.303
	Gilts	0.392	0.412	0.406	0.417	0.424				0.005	0.751
SID Lys/gain, g/kg	Boars	16.5	18.1	19.5	21.1	22.8	0.36	0.001	0.757	<0.001	0.779
	Gilts	16.6	18.2	21.0	22.9	24.8				<0.001	0.875
Feed cost/gain, €/kg	Boars	0.568	0.557	0.547	0.547	0.550	0.0100	0.004	0.594	0.146	0.323
	Gilts	0.570	0.561	0.588	0.592	0.599				0.007	0.811

^1^A total of 780 pigs (Pietrain NN × (Landrace × Large white) in pens of 13, with six replicates per treatment and sex. ^2^Calculated SID Lys:NE. ^3^Interaction between sex and the linear response to SID Lys:NE. ^4^Interaction between sex and the quadratic response to SID Lys:NE.

In the second period (Table 5), from 89 to 106 kg BW, there were no significant interactions between sex and the linear or quadratic response to SID Lys:NE. Only a tendency for a different linear response to increased SID Lys was reported for SID Lys per kg gain. Neither boars (*P* = 0.328) nor gilts (*P* = 0.764) showed a linear increase in ADG when SID Lys:NE was increased. Moreover, boars’ G:F linearly increased (*P* = 0.003) whereas gilts just showed a tendency (*P* = 0.098). The difference between the response in ADG and G:F could be partly related to a tendency for ADFI to decrease in both boars (*P* = 0.052) and gilts (*P* = 0.089). As in the previous period, increasing levels of SID Lys:NE increased SID Lys per kg gain linearly in both boars and gilts (*P* < 0.001). However, gilts tended to show a greater linear increase than boars (*P* = 0.100).

**Table 5.** Interactive effects between standardized ileal digestible lysine to net energy ratio (SID Lys:NE) and sex on growth performance and economics of finishing boars and gilts in the Period 2 (89 to 106 kg)^1^

Item		SID Lys:NE, g/Mcal^2^					SEM	*P*-value			
		2.64	3.05	3.46	3.86	4.27		L × S^3^	Q × S^4^	Linear	Quadratic
ADG, kg	Boars	0.875	0.918	0.933	0.906	0.920	0.0254	0.630	0.505	0.328	0.296
	Gilts	0.783	0.836	0.781	0.802	0.812				0.764	0.915
ADFI, kg	Boars	2.27	2.25	2.29	2.16	2.17	0.044	0.853	0.971	0.052	0.525
	Gilts	2.21	2.34	2.18	2.17	2.17				0.089	0.559
G:F	Boars	0.386	0.408	0.408	0.420	0.425	0.0092	0.314	0.494	0.003	0.535
	Gilts	0.356	0.357	0.360	0.372	0.374				0.098	0.727
SID Lys/gain, g/kg	Boars	16.9	18.5	20.9	22.6	24.8	0.55	0.100	0.537	0.000	0.773
	Gilts	18.4	21.1	23.7	25.8	28.2				0.000	0.559
Feed cost/gain, €/kg	Boars	0.580	0.568	0.585	0.586	0.600	0.0154	0.409	0.476	0.236	0.556
	Gilts	0.631	0.649	0.665	0.667	0.680				0.021	0.674

^1^A total of 780 pigs (Pietrain NN × (Landrace × Large white) in pens of 13, with six replicates per treatment and sex. ^2^Calculated SID Lys:NE. ^3^Interaction between sex and the linear response to SID Lys:NE. ^4^Interaction between sex and the quadratic response to SID Lys:NE.

Table 6 provides the experimental results on BW and growth performance for the *Overall* period. The top half of the table shows that there was no evidence that increasing SID Lys:NE resulted in a greater linear increase in BW of boars at day 22 (*P* = 0.712) or day 41 (*P* = 0.591). However, when SID Lys:NE was increased boars showed a linear increase in BW at day 41 (*P* = 0.037), but the increase was not significant for gilts (*P* = 0.173). Regarding overall growth performance, there was evidence of a different response to SID Lys:NE between boars and gilts for most variables studied. Average daily gain tended to show a greater linear increase for boars than gilts (*P* = 0.087). Consistently, boars’ ADG increased linearly (*P* < 0.001), whereas there was no significant effect for gilts (*P* = 0.103). As in *Period 1*, boars showed a greater linear increase in G:F (*P* = 0.003), whereas gilts showed an increase in SID Lys per kg gain (*P* < 0.001) and feed cost per kg gain (*P* = 0.005) when SID Lys:NE was increased.

**Table 6.** Interactive effects between standardized ileal digestible lysine to net energy ratio (SID Lys:NE) and sex on body weight, growth performance, and economics of finishing boars and gilts in the overall experiment (70 to 106 kg)^1^

		SID Lys:NE, g/Mcal^2^					SEM	*P*-value			
		2.64	3.05	3.46	3.86	4.27		L × S^3^	Q × S^4^	Linear	Quadratic
Body weight, kg											
Day 0	Boars	70.4	70.7	70.4	70.4	70.4	0.79	0.563	0.844	0.879	0.912
	Gilts	68.4	68.6	68.5	68.8	69.1				0.506	0.867
Day 22	Boars	88.7	90.9	91.0	90.9	91.8	1.00	0.712	0.693	0.055	0.461
	Gilts	86.5	87.9	87.4	88.2	88.6				0.156	0.857
Day 41	Boars	105.3	108.6	108.5	108.2	109.2	1.11	0.591	0.492	0.037	0.259
	Gilts	101.3	103.8	102.2	103.2	104.0				0.173	0.871
Growth performance											
ADG, kg	Boars	0.851	0.918	0.933	0.921	0.946	0.0149	0.087	0.348	<0.001	0.049
	Gilts	0.804	0.861	0.822	0.845	0.851				0.103	0.497
ADFI, kg	Boars	2.18	2.23	2.21	2.11	2.14	0.038	0.651	0.878	0.084	0.353
	Gilts	2.15	2.23	2.14	2.14	2.13				0.268	0.476
G:F	Boars	0.390	0.412	0.423	0.437	0.444	0.0051	0.003	0.326	<0.001	0.196
	Gilts	0.375	0.386	0.384	0.396	0.400				<0.001	0.925
SID Lys/gain, g/kg	Boars	16.7	18.2	20.1	21.8	23.7	0.27	<0.001	0.465	<0.001	0.663
	Gilts	17.3	19.5	22.2	24.0	26.3				<0.001	0.549
Feed cost/gain, €/kg	Boars	0.573	0.561	0.565	0.564	0.572	0.0074	0.005	0.314	0.965	0.210
	Gilts	0.596	0.600	0.622	0.622	0.634				<0.001	0.867

^1^A total of 780 pigs [Pietrain NN × (Landrace × Large white)] in pens of 13, with six replicates per treatment and sex. ^2^Calculated SID Lys:NE. ^3^Interaction between sex and the linear response to SID Lys:NE. ^4^Interaction between sex and the quadratic response to SID Lys:NE.

The interactive effects between sex and SID Lys:NE on carcass characteristics are reported in Table 7. There was no significant interaction between sex and the linear (*P* = 0.151) or quadratic (*P* = 0.135) effect of SID Lys:NE on HCW. Nevertheless, boars’ HCW increased linearly (*P* = 0.027), whereas there was no evidence of an increase for gilts (*P* = 0.821). Similarly, the CY of gilts was linearly (*P* = 0.042) reduced when SID Lys:NE was increased, whereas the CY of boars was not (*P* = 0.904); however, there was no significant interaction in the linear response (*P* = 0.180). In accordance with the results on growth performance, boars had a greater linear increase in lean tissue and a reduction in fat content when SID Lys:NE was increased. The interaction between sex and the linear effect of SID Lys:NE was significant for both CL (*P* = 0.016) and BFT (*P* = 0.026) but not for HFT (*P* = 0.230). Regarding boars, increasing SID Lys:NE led to a linear increase in CL (*P* < 0.001), and a linear decrease in BFT (*P* < 0.001) and HFT (*P* = 0.002). However, there was only a quadratic (*P* = 0.044) effect of SID Lys:NE on BFT, but no evidence of an effect on CL or BFT for gilts (*P* ≥ 0.129).

**Table 7.** Interactive effects between standardized ileal digestible lysine to net energy ratio (SID Lys:NE) and sex on carcass weight and composition of finishing boars and gilts (70 to 106 kg)^1^

		SID Lys:NE, g/Mcal^2^					SEM	*P*-value			
		2.64	3.05	3.46	3.86	4.27		L × S^3^	Q × S^4^	Linear	Quadratic
HCW, kg	Boars	87.2	90.0^6^	90.6^6^	89.6	90.9^6^	0.95	0.151	0.135	0.027	0.197
	Gilts	86.7	87.1^6^	85.7	86.5	87.3^6^				0.821	0.405
CY, %^5^	Boars	77.2^7^	77.0	77.4^6^	77.2^7^	77.1^6^	0.28	0.180	0.836	0.904	0.649
	Gilts	79.2	78.5	79.0^6^	78.6	78.3^7^				0.042	0.869
CL, %^5^	Boars	63.7	63.5^7^	64.8^6^	64.8	65.5^6^	0.31	0.016	0.320	<0.001	0.688
	Gilts	63.1	63.5	63.5^7^	63.8	63.6				0.181	0.305
BFT, mm^5^	Boars	14.7^7^	14.7	13.8^6^	13.8^7^	13.3^6^	0.30	0.026	0.142	<0.001	0.933
	Gilts	15.1^7^	14.6	14.4^6^	14.3	14.9^7^				0.396	0.044
HFT, mm^5^	Boars	9.25	9.30^6^	8.60^6^	8.69	8.28^7^	0.23	0.174	0.484	0.002	0.903
	Gilts	10.61	10.37	10.17^6^	10.12	10.18				0.129	0.377

^1^A total of 780 finishing pigs [Pietrain NN × (Landrace × Large white)] in pens of 13 were used in a 41 days growth trial, with two periods, including six pens per treatment and sex. All pigs reaching marketing were transported to a commercial slaughter and packing plant (Patel, Spain) to collect individual data on carcass composition, but only 650 pigs could be completely traced. Observations per pen were weighted using the number of pigs per pen for which carcass data was available. ^2^Calculated SID Lys:NE. ^3^Interaction between sex and the linear response to SID Lys:NE. ^4^Interaction between sex and the quadratic response to SID Lys:NE.

^5^Adjusted for HCW. ^6^SEM was 0.97 for HCW, 0.29 for CY, 0.33 for CL, 0.31 for BFT, and 0.24 for HFT in the indicated means. ^7^SEM was 0.27 for CY, 0.32 for CL, 0.029 for BFT, and 0.25 for HFT in the indicated means.

### Estimation of SID Lys:NE Requirements of Boars and Gilts

Dose–response BLL models were fitted to predict ADG for each sex and period. For the initial period, models could be fitted for each sex, and different responses were found. For boars, two models reported similar fits (BIC = 316.1 and 316.5). The best fitting model for boars was as follows:

$$\begin{array}{ll} ADG\;\left(g\right)= & 367+286\times ADFI\;(kg)\\ & -92.6\times (4.05-SID\;Lys:NE) \end{array}$$

if SID Lys:NE ≤ 4.05, and when SID Lys:NE > 4.05 then

$$\begin{array}{l} ADG\;\left(g\right)=367+286\times ADFI\;(kg) \end{array}$$

in which ADG was transformed to gram to facilitate the fitting process, and maximum growth was reached at 4.05 g SID Lys/Mcal NE [95% CI: (3.56, 4.54)]. Whereas the other model gave an optimum for maximum growth of 3.71 g SID Lys/Mcal NE [95% CI: (3.30, 4.12)].

The best fitting model for gilts (BIC = 307.4) in the first period was modified because the intercept of the model was insignificant as it was close to 0; therefore, the model was fitted without an intercept as follows:

$$\begin{array}{ll} ADG\;(g)= & 415\times ADFI\;(kg)\;98.9\\ & \times (3.13-SID\;Lys:NE) \end{array}$$

if SID Lys:NE ≤ 3.13, and when SID Lys:NE > 3.13, then

$$\begin{array}{l} ADG\;(g)=415\times ADFI\;(kg) \end{array}$$

with maximum growth reached at 3.13 g SID Lys/Mcal NE [95% CI: (2.74, 3.51)].

It was not possible to adjust a model in the second period for gilts. Fit was possible for boars, but the slope (*U*) was not significant, and therefore, the models were not considered.

Finally, models for the overall period could be fitted for both boars and gilts. Like in the first phase, for boars there were two models with similar fitting (BIC = 289.2 and 290.3). The best fitting model was the following:

$$\begin{array}{ll} ADG\;(g)= & 405+249\times ADFI\;(kg)-92.4\\ & \times (3.63-SID\;Lys:NE) \end{array}$$

if SID Lys:NE ≤ 3.63, and when SID Lys:NE > 3.63, then

$$\begin{array}{l} ADG\;\;\;(g)=405+249\times ADFI\;\;\;(kg) \end{array}$$

in which maximum growth was reached at 3.63 g SID Lys/Mcal NE [95% CI: (3.32, 3.94)]. Contrastingly, the maximum growth in the model with just slightly the worst fit was reached at 4.01 g SID Lys/Mcal NE [95% CI: (3.60, 4.42)].

**Figure 2.**
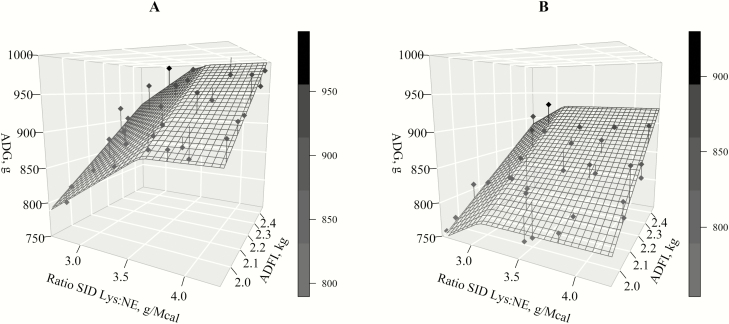
Fitted broken-line linear models to predict ADG as a function of increasing the standardized ileal digestible lysine to net energy ratio (SID Lys:NE) and ADFI. Maximum ADG was estimated for boars (A) at 3.63 g SID Lys/Mcal NE [95% CI: (3.32, 3.94) g SID Lys/Mcal NE] and for gilts (B) at 3.10 g SID Lys/Mcal NE [95% CI: (2.74, 3.47) g SID Lys/Mcal NE].

For gilts, the model that best described growth (BIC = 296.2) depending on the SID Lys:NE and feed intake was as follows:

$$\begin{array}{ll} ADG\;\left(g\right)= & 270+267\times ADFI\;(kg)-82.3\\ & \times (3.10-SID\;Lys:NE) \end{array}$$

if SID Lys:NE ≤ 3.10, and when SID Lys:NE > 3.10, then

$$\begin{array}{l} ADG\;\left(g\right)=270+267\times ADFI\;(kg) \end{array}$$

in which maximum growth was achieved at 3.10 g SID Lys/Mcal NE [95% CI: (2.74, 3.47)]. The two best fitting models in the overall period are shown in Fig. 2.

## DISCUSSION

In the context of an increasing production of entire males, this study focused on determining whether boars and gilts respond differently to SID Lys:NE, and modeling their response to optimize growth performance. Consistent with the literature, this study observed that boars have a greater ADG and G:F than gilts and a similar ADFI. The greater ADG of boars has been related to a higher potential for lean tissue deposition (Hendriks and Moughan, 1993; Rikard-Bell et al., 2013b), starting around 50 to 70 kg BW (Whittemore et al., 1988; Giles et al., 2009; Carabús et al., 2017). Studies analyzing growth data have observed that gilts’ ADG compared with boars’ ADG starts to decline around 40 to 70 kg BW (Campbell et al., 1988; Quiniou et al., 2010; Cámara et al., 2014). In addition, boars greater G:F might be explained by their greater growth potential and considering that there was no evidence of differences in ADFI (O’Connell et al., 2006; Cámara et al., 2014). Moreover, in agreement with the literature, boars were generally leaner than gilts (Cámara et al., 2014; Carabús et al., 2017) and had a reduced carcass yield, which might be partly attributed to the removal of the testicles (Gispert et al., 2010). The differences in BFT were in agreement with Rikard-Bell et al. (2013b). However, the small difference compared with HFT might explain why other authors did not obtain this result (Gispert et al., 2010; Trefan et al., 2013). Finally, as genetic improvements during the last decades could be responsible for part of the differences between studies, reference values should be carefully considered.

### Differential Response to SID Lys:NE

These results confirmed the different productive performance response of boars and gilts to SID Lys:NE. As mentioned in the introduction, some works have already studied this response in recent decades (Campbell et al., 1988; Van Lunen and Cole, 1996; King et al., 2000; O’Connell et al., 2006; Moore et al., 2013; Rikard-Bell et al., 2013a). However, so far there is no single experiment in the literature that compares the linear or quadratic trends between sexes in one statistical model. Most authors just report the effect of dietary lysine on each sex. As recently shown by Aymerich et al. (2020), comparing the linear or quadratic response in a single model gives an indication of how certain we can be that the effect of SID Lys:NE differs between categories of a factor. However, being linear or quadratic should be considered carefully as this could be strongly influenced by the range of SID Lys:NE in which the experiment evaluated the effects on pig performance. Throughout the discussion, as most experiments used a ratio between SID Lys and metabolizable energy (ME) or digestible energy (DE), a ratio of 0.71 between NE and DE and 0.74 between ME and NE is assumed to make comparisons with the results from other studies possible (Noblet and van Milgen, 2004).

The current study reports evidence that boars and gilts respond differently to increasing SID Lys:NE, although due to the great variability only a tendency was found for ADG. The response of boars up to a higher SID Lys:NE is explained because more lysine is needed to reach their greater protein deposition potential. Thus, as the experiment was in accordance with previous literature that shows no significant differences in ADFI between boars and gilts (O’Connell et al., 2006; Cámara et al., 2014), boars responded to diets that are more concentrated in dietary lysine to maximize performance. The response for ADG was different to Moore et al. (2013), who found linear and quadratic effects of dietary lysine in both finishing boars and gilts in a SID Lys:NE range from 2.3 to 4.6 g/Mcal. However, Rikard-Bell et al. (2013a) reported that boars have a response in ADG up to a higher SID Lys:NE than gilts in a range from 2.4 to 4.2 g SID Lys/Mcal NE. It is possible that the SID Lys:NE range in our study was not sufficient to significantly limit growth in gilts. Using a lower bottom SID Lys:NE boundary might have provided a better understanding of the form of the different responses between sexes.

A reduction in ADG and G:F at the highest lysine levels has been reported in other studies (Van Lunen and Cole, 1996; O’Connell et al., 2006; Moore et al., 2013). This outcome differs from the findings presented here, in which ADG did not decrease at lysine levels above the requirement. A hypothesis is that the reduction in growth at high lysine levels is explained by an increase in heat production when CP levels are above the requirement for growth. Le Bellego et al. (2001) showed that reducing CP while using synthetic AA to fulfill ideal protein requirements increased the efficiency of ME and NE use. Otherwise, excess AA has to be deaminated, which has an energy cost (Bender, 2012). Hence, it could conceivably be hypothesized that the high CP diets used in the high lysine treatments of some of the works reviewed, most of them above 17% (Campbell et al., 1988; Van Lunen and Cole, 1996; King et al., 2000; O’Connell et al., 2006; Moore et al., 2013), might explain why there was a reduction in feed efficiency and ADG.

The carcass composition results corroborated the findings of Moore et al. (2013), reporting a linear effect of SID Lys:NE only on the backfat thickness of boars, while there was no linear effect on gilts. Similarly, Lambe et al. (2013) showed that fatness of entire male Pietrain sire-line pig carcasses increased only when the lysine content of the diet was reduced, and not for a lower CP. In addition, Rikard-Bell et al. (2013b) showed that when SID Lys:NE was increased from 3.3 to 3.8 g/Mcal, there was only a significant increase in boars’ lean tissue deposition. The different linear effect between sexes reported in this experiment supports the different linear responses observed in growth performance results. It confirmed that the greater response of boars to increasing SID Lys:NE entailed an increase in carcass leanness. Therefore, at the lower ratios, SID Lys intake was limiting boars’ protein deposition, and consequently, energy was used for fat deposition, increasing BFT. However, the results also suggest that gilts cannot increase lean deposition when offered more SID Lys. Nevertheless, the inconsistency with some of the available literature might be a result of different statistical analyses, experimental diet formulation strategies, or genetic lines.

### Modeling SID Lys:NE Requirements

Several studies have aimed to model the results from a dose–response trial to determine an optimum for different parameters such as ADG or G:F. It is widely acknowledged that the model used (broken-line linear, quadratic polynomial, broken-line quadratic) is a major factor in determining the optimum level (Pesti et al., 2009). In the present study, BLL was preferred because quadratic trends were not reported for almost any variable. The objective of the fitted models was to describe the differential response to SID Lys:NE on ADG of boars and gilts reported in the results section. Although models could be fitted, having a lower bottom SID Lys:NE level would have made the fitting process easier. For instance, Robbins et al. (2006) suggested that a minimum of four points below the requirement are needed to fit the shape of a quadratic response.

Including ADFI as a covariate in the model, which explained 0.40 to 0.60 of the ADG variability, enabled fitting a BLL model to predict the ADG of gilts depending on SID Lys:NE, although there was only one level that theoretically limited growth. As a result, both dose–response broken-line linear models for gilts and boars could be fitted for *Period 1* and *Overall*, and the requirements between sexes were compared using the CI of the break point, the SID Lys:NE at which ADG was maximized. Seventy to 89 kg BW boars showed a response in ADG up to a higher SID Lys:NE compared with gilts. Nevertheless, in the *Overall* period, the CIs were overlapped, considering the best fitting model; however, if the boars’ second best fitting model, with a similar BIC, is considered, then the CI would not be overlapped because it reported a higher break point. Therefore, in agreement with the different linear effects of SID Lys:NE on growth performance, boars required greater dietary SID Lys:NE, mainly for the period 70 to 89 kg, to maximize growth performance.

The greater requirements for boars compared with gilts corroborate the findings of some previous studies (O’Connell et al., 2006; Rikard-Bell et al., 2013a), contradict other studies (Van Lunen and Cole, 1996; King et al., 2000), and some studies did not show relevant differences (O’Connell et al., 2006; Moore et al., 2013). However, most of these studies did not report a CI for the requirement estimates, and thus, doubts arise about comparisons of requirements of the two sexes. For instance, the requirement for boars to optimize ADG from 70 to 106 kg (3.63 to 4.01 g SID Lys/Mcal NE) was slightly lower to that reported by Rikard-Bell et al. (2013a; >4.24 g SID Lys/Mcal NE), whereas it was slightly higher than that reported by Moore et al. (2016; 3.4 g SID Lys/Mcal NE) using BLL models. The requirement for gilts, although low compared with some studies (O’Connell et al., 2006; Shelton et al., 2011; Moore et al., 2013), was similar to Rikard-Bell et al. (2013a; 3.2 g SID Lys/Mcal NE) and greater than Main et al. (2008; 2.6 g SID Lys/Mcal NE).

These results suggest that the requirements for boars were 117% higher than gilts or even more, whereas the review in Dunshea et al. (2013) suggested that they were only 108%. However, Williams et al. (1984) suggested that the requirements for boars might be 125% the requirements of barrows, considering that barrows and gilts have similar requirements (Main et al., 2008). The differences in requirements might be partly due to the potential ADG, which in this study was 11% greater for boars (940 vs. 844 kg), assuming a 2.15 kg ADFI. However, data from studies in which the SID Lys:NE of the diets fed to pigs could have been limiting boars’ growth should be used carefully to model requirements. Furthermore, as suggested by Lerman and Bie (1975), dose–response growth models need to be linked to lysine cost models to determine exactly which is the best level from an economic standpoint. The present models are a first step for determining which are the economically optimal diets for each sex. Nevertheless, the results already suggest that feeding gilts with an optimal diet for boars may increase the production cost of gilts.

In conclusion, the different responses in growth performance and carcass composition of boars and gilts to increasing SID Lys:NE along with the modeling outcomes indicate that finishing boars (70 to 110 kg) have a greater SID Lys:NE requirement than gilts to maximize growth performance and carcass leanness. This is explained by the greater protein deposition and therefore growth potential of boars starting from 50 to 70 kg to market weight, when there are no evident differences in ADFI. Therefore, the present work suggests that feeding boars and gilts diets with different SID Lys:NE during the finishing period might be beneficial from both the performance and cost-return perspectives.

*Conflict of interest statement*. The authors declare no conflict of interest.
